# The ascending limb of the cocaine unit dose–response function in rats as an experimental artifact

**DOI:** 10.1038/s41598-023-43506-y

**Published:** 2023-10-03

**Authors:** Jhanvi N. Desai, Luis E. Tron Esqueda, Andrew B. Norman

**Affiliations:** Department of Pharmacology and Systems Physiology, University of Cincinnati College of Medicine, Cincinnati, OH USA

**Keywords:** Neuroscience, Diseases of the nervous system, Addiction

## Abstract

The cocaine unit dose–response function is an inverted U with the ascending and descending limbs representing the positive and rate limiting cocaine effects, respectively. Higher fixed ratio (FR) schedules and/or time-out periods make the ascending limb more prominent. Alternatively, a pharmacokinetic/pharmacodynamic interaction theory demonstrates that cocaine-induced lever pressing occurs only when cocaine levels are within a range termed the compulsion zone. The inter-injection intervals of self-administration increase with cocaine unit dose because of the longer time required to eliminate higher doses. However, this theory has not been applied to high FR schedules. Rats acquired cocaine self-administration on a FR1 schedule and then were changed to sessions that started with both FR1 and then FR50 over a range of unit doses with a set number of self-administrations allowed for each dose. On FR1, rats completed the maximum number of injections at all but the lowest unit dose. In contrast, on FR50 the proportion of the permitted injections increased as a function of unit dose. However, this ascending limb was the result of averaging data from sessions where rats completed or failed to complete the allowed number of injections. Rats completed all injections when cocaine levels were maintained in the compulsion zone. The FR50 schedule and low unit doses decreased this probability of maintaining cocaine levels in the compulsion zone when the rate of cocaine elimination exceeded the rate of cocaine input during the time required to complete the 50 presses. It is concluded that the ascending limb is an experimental artifact and that the entire dose–response function and the FR50-induced increase in inter-injection intervals are explained in terms of the compulsion zone theory of cocaine self-administration behavior.

## Introduction

It is considered axiomatic that cocaine self-administration behavior by animals is an example of operant behavior theory and that cocaine acts as a reinforcer^1,2^. Consequently, cocaine self-administration behavior should be shaped by schedules of drug delivery^3,4^. Positive reinforcers typically increase the rate of responding^5^. For a typical graded agonist-induced response, the magnitude of response increases as a function of the agonist concentration or dose as increasing numbers of receptors underlying the response are occupied by the agonist until a maximum response is reached as the receptor population approaches saturation by the agonist^6^. The initial studies of cocaine self-administration behavior in rats reported that as the cocaine unit dose (the amount of each cocaine injection) increased, the rate of cocaine self-administration decreased^1^. Positive reinforcers typically increase the rate of responding, and subsequent studies of the cocaine unit dose-rate of responding relationship reported that at low doses response rates do increase up to a maximum rate and further increases in unit dose result in a gradually decreasing rate of responding^2,7^. These dose-dependent increases or decreases in the response rate are referred to as the ascending and descending limbs of the cocaine dose response curve, respectively^2^. Therefore, the inverted U shape of the dose–effect curve reflects an interaction between cocaine’s positive reinforcing effect, which increase response rates and other rate limiting effects which decrease rates of operant responding. There has been debate concerning the mechanisms that limit rates of responding for cocaine, though consensus has yet to be reached. It is also reported that the schedule of cocaine delivery influences the position of the ascending limb, the peak and the descending limb of the cocaine dose–effect curve^2,8^.

It has also long been recognized that cocaine is a drug and cocaine’s effects should be exerted according to the principles of pharmacology^9^ via dopamine neurotransmission^10–12^. It has been demonstrated that cocaine induces lever pressing behavior only when cocaine levels are above the priming/remission threshold and below the satiety threshold, a range termed the compulsion zone^13^. In contrast to the operant behavior approach, this pharmacological model has been applied to cocaine self-administration behavior and demonstrates that the cocaine unit dose-rate of injections during maintained self-administration behavior is the result of a pharmacokinetic/pharmacodynamic interaction^14^. The decrease in the rate of injections at higher unit doses is due to the increasing time required for the higher cocaine levels to fall back to a minimum cocaine level in the body that is referred to as the satiety threshold^14^. This time is dependent on the pharmacokinetics of cocaine in rats^15^. This model of cocaine self-administration is consistent with the pharmacokinetic/pharmacodynamic regulation of amphetamine self-administration reported in rats^16^. The satiety threshold is assumed to represent the activation of a set number of dopamine receptors that is constant and independent of the cocaine unit dose^17^. Thus, in this approach the dose–effect function represents a dose-duration of response and not a dose-magnitude of response function^18^. There is no requirement for a biphasic dose–effect function in this model. Indeed, when the rate of self-administration over a range of low cocaine unit doses was investigated, there was no evidence that the rate of self-administration increased as the unit dose increased, only a decrease in the rate of self-administration was observed^13^. However, the failure to replicate the ascending limb of the cocaine dose–effect function in rats only used the simplest, FR1, schedule of cocaine delivery^13^, and the ascending limb is not as prominent when using an FR1 schedule^19^. Given that the inverted U shaped dose–response curve is universally accepted and experimentally demonstrated, but the compulsion zone theory does not predict any ascending limb, we investigated whether high FR schedules in the context of the compulsion zone could resolve this paradox. Here we directly compared the effect of FR1 and a considerably higher FR50 schedule of cocaine delivery on the cocaine unit dose-rate of self-administration function in rats.

## Results

### Effect of FR schedule on Inter-injection intervals during cocaine self-administration in FR1/FR50 sessions

**Figure 1:**
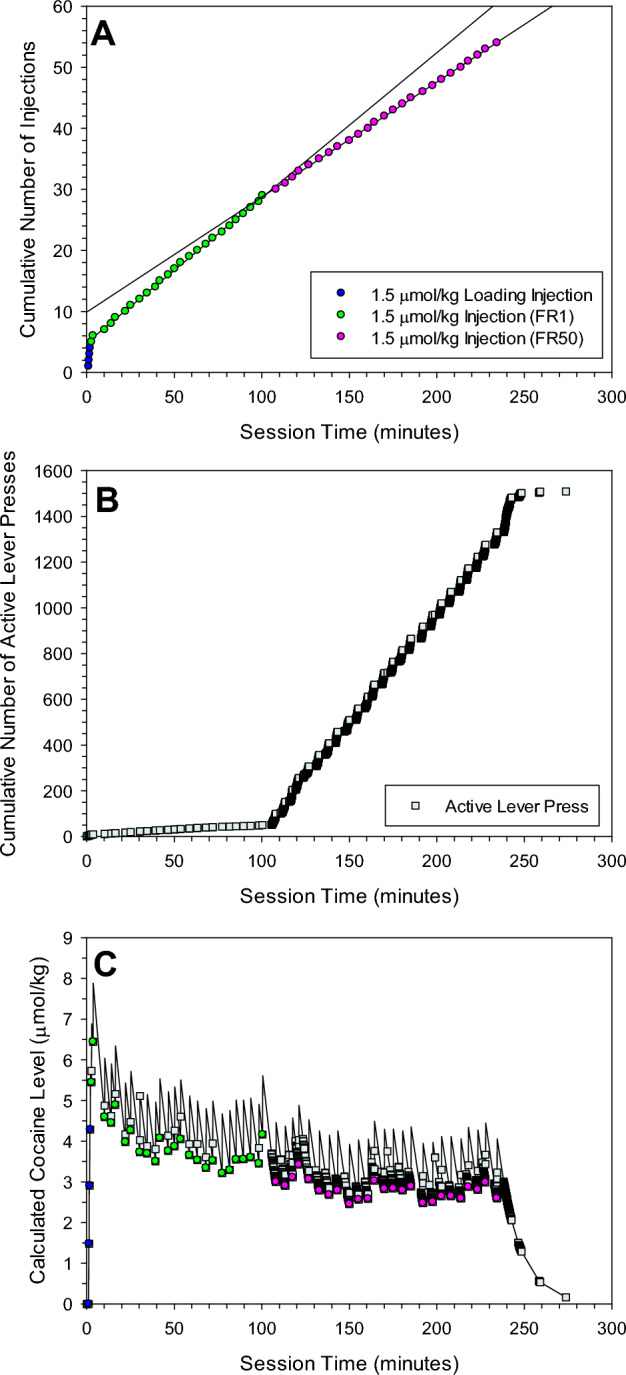
Cumulative record of injections (**A**), active lever presses (**B**), and the calculated levels of cocaine at the time of each event (**C**) during a representative session. Once rats acquired self-administration behavior on FR50 schedule, rats were on FR1/FR50 sessions in which the unit dose was one of 0.1, 0.3, 0.5, 0.75, 1.5 and 3 µmol/kg, and the number of injections allowed for each FR phase was 225, 75, 60, 50, 25 and 15 respectively. This figure shows a representative FR1/FR50 session at 1.5 µmol/kg cocaine unit dose. After 4 loading injections of the standard loading dose (1.5 µmol/kg), 25 unit doses were administered for each schedule, and the rat displayed regular cocaine self-administration on each schedule. After access to cocaine was terminated, active lever presses had no consequence but were recorded until lever pressing stopped.

**Figure 2:**
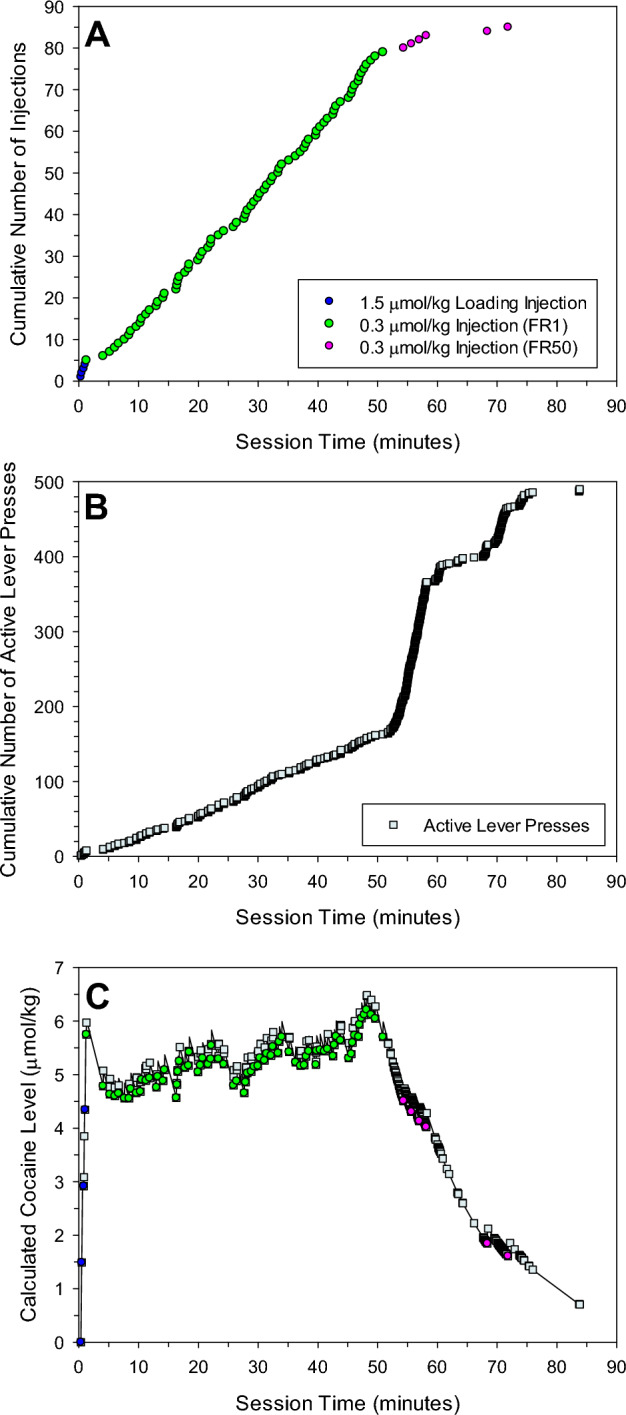
Cumulative record of injections (**A**), active lever presses (**B**), and the calculated levels of cocaine at the time of each event (**C**) during a FR1/FR50 representative session at 0.3 µmol/kg unit dose. A maximum of 75 injections was allowed for both FR1 and FR50 schedule at this unit dose. In this session, the rat self-administered 4 loading injections of the standard loading dose (1.5 µmol/kg), then completed 75 injections on FR1, but only completed 6 injections on FR50 schedule before meeting the criterion used to end the session. Cocaine self-administration was regular on FR1. On the FR50 schedule, cocaine levels kept decreasing as the rat performed the 50 lever presses to get the next injection, and the resultant injection of 0.3 µmol/kg dose was not enough to maintain cocaine levels in the range maintained previously during cocaine self-administration on FR1.

Inter-injection intervals were regular on FR1 as demonstrated by a lack of deviation from the linear regression line (Fig. 1A). When the schedule was switched from FR1 to FR50, the intervals were again regular, but were longer (Fig. 1A). Inter-injection intervals on FR1 increased as the unit dose was increased (compare Figs. 1A and 2A, Supplemental Table S1). Similar to FR1, on FR50 the inter-injection intervals also increased as a function of the unit dose. Furthermore, inter-injection intervals on FR50 were consistently longer than the FR1 values at the same unit dose (Supplemental Table S1). Self-administration at lower cocaine unit doses was not always maintained on FR50 (Table 1, Fig. 2A).

**Table 1:** Number of injections self-administered on FR1 and FR50 schedule at different unit doses.

Unit Dose (µmol/kg)	Maximum Number of Injections	Number of injections completed		Number (%) of the 15 sessions where all injections were completed	
		FR1	FR50	FR1	FR50
0.1	225	145 ± 39	9 ± 3*	8 (53%)	0
0.3	75	75 ± 0	32 ± 14*	15 (100%)	5 (33%)
0.5	60	60 ± 0	37 ± 10*	15 (100%)	7 (47%)
0.75	50	50 ± 0	33 ± 6*	15 (100%)	7 (47%)
1.5	25	25 ± 0	25 ± 0	15 (100%)	15 (100%)
3	15	15 ± 0	15 ± 0	15 (100%)	15 (100%)

Values show the mean ± SEM from 5 rats. The number of sessions where values were obtained was 15 except for 0.1 µmol/kg where rats did not complete the FR1 component and there was no corresponding FR50 measurement.

*Significantly different (*P* < 0.05) than the corresponding FR1 value.

### Effect of FR schedule on lever presses during cocaine self-administration in FR1/FR50 sessions

In contrast to the decrease in rate of injections at a unit dose on FR50 (Figs. 1A, 2A, Supplemental Fig. S1A), lever pressing activity increased on FR50 relative to FR1 (Figs. 1B, 2B, Supplemental Fig. S1B). There were few additional lever presses between injections on FR1, and these occurred during the time-out period. However, as required, there were at least 50 lever presses between each injection on FR50, in addition to any time-out lever presses. The rate of lever pressing for the 50 required lever presses was variable and the duration of these 50 presses ranged from approximately 1–1.8 min.

### Effect of FR50 on the proportion of the maximum number of cocaine injections allowed and probability of completing a session at different unit doses

**Figure 3:**
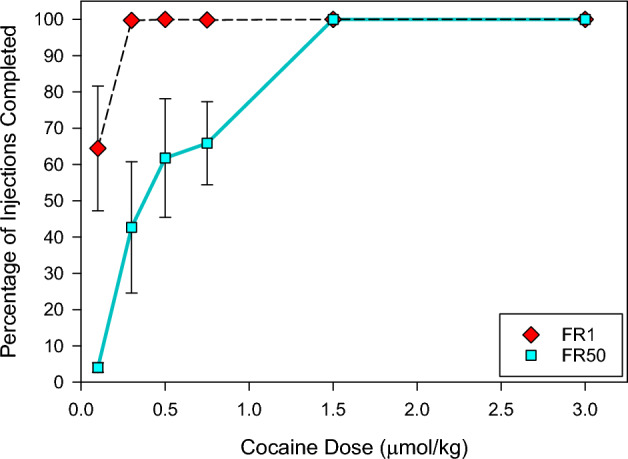
Percentage of injections completed during cocaine self-administration on FR1 and FR50 schedule in FR1/FR50 sessions at a unit dose. The unit dose was one of 0.1, 0.3, 0.5, 0.75, 1.5 or 3 µmol/kg, and the number of injections allowed for each FR phase was 225, 75, 60, 50, 25 and 15 respectively. Data points show Mean ± SEM from 5 rats. There were 3 sessions per rat.

Rats completed 100% of the allowed injections on FR1 at 0.3, 0.5, 0.75, 1.5, and 3 µmol/kg unit doses, and 64% of the allowed injections at 0.1 µmol/kg unit dose (Fig. 3). In the 8/15 sessions at 0.1 µmol/kg unit dose where rats completed all 225 allowed injections on FR1, they resumed cocaine self-administration when switched to the FR50 schedule. In these sessions, an average of 4% of the allowed injections were completed. The percentage of allowed injections increased from 43 to 66% over the 0.3, 0.5 and 0.75 µmol/kg unit doses (Fig. 3).

Similarly, the probability of completing all the allowed injections in a session on the FR50 schedule in the 15 sessions at each dose ranged from 0% at 0.1 µmol/kg to less than 50% at 0.5 and 0.75 µmol/kg (Table 1). At the 1.5, and 3 µmol/kg unit dose, rats completed all of the allowed injections on FR50 schedule in all sessions (Table 1, Fig. 3).

### Effect of averaging data on FR50 schedule from sessions where all injections were self-administered and sessions where rats failed to self-administer all injections

**Figure 4:**
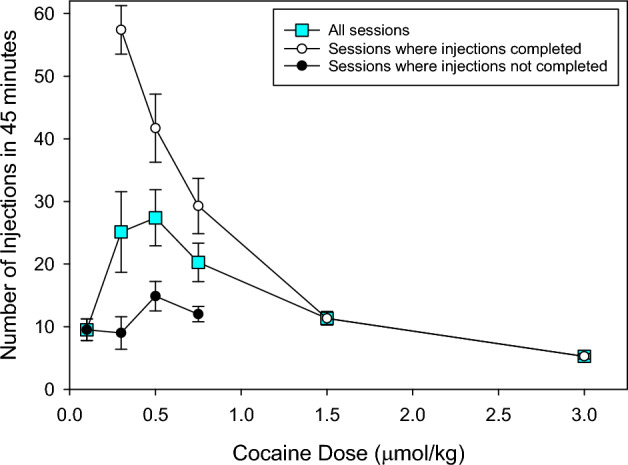
The ascending limb is dependent on the inclusion of data from sessions in which rats failed to self-administer all allowed injections. Data points represent Mean ± SEM number of injections on FR50 schedule in 45 min from 15 sessions from 5 rats at each unit dose. The number of sessions where rats self-administered all allowed injections on FR50 schedule is shown in Table 1.

When data from all sessions that started cocaine self-administration on FR50 schedule were averaged the rate of injections (number in 45 min) completed, first increased and then decreased as a function of the cocaine unit dose (Fig. 4, Supplemental Fig. S1A). Similarly, in sessions where rats did not self-administer all of the allowed injections on FR50 schedule, rate of injections appeared to first increase and then decrease over the 0.1, 0.3, 0.5 and 0.75 µmol/kg unit doses (Fig. 4). Conversely, in sessions where rats self-administered all of the allowed injections on FR50, the rate of injections was inversely proportional to cocaine unit dose across the entire dose range (Fig. 4).

### Effect of FR schedule in FR1/FR50 sessions on calculated cocaine levels at the time of injection during cocaine self-administration

Cocaine levels at the time of injection was consistent and within the range of (4.9–6.1 µmol/kg) during cocaine self-administration on FR1 schedule at all unit doses (for examples see Figs. 1C, 2C).

At the 1.5 and 3 µmol/kg unit doses where rats completed all the allowed number of injections on FR50 schedule, the injections on FR50 occurred at a lower level compared to that on FR1 (Fig. 1C). The mean ± SEM level of cocaine at the time of injections at 1.5 µmol/kg unit dose across all sessions was 6.1 ± 0.1 µmol/kg on FR1 compared to 4.5 ± 0.1 µmol/kg on FR50. The mean ± SEM level of cocaine at the time of injections at 3 µmol/kg unit dose across all sessions was 4.9 ± 0.1 µmol/kg on FR1 compared to 3.6 ± 0.1 µmol/kg on FR50.

At 0.3, 0.5, and, 0.75 µmol/kg unit doses the cocaine levels at the time of injections on FR50 schedule had a larger range of 3.6–5.2 µmol/kg (data not shown). On FR50 schedule, rats failed to complete all of the allowed number of injections at the 0.3, 0.5, and 0.75 µmol/kg unit doses in 10, 8, and 8 of the 15 sessions respectively. In these sessions, cocaine levels decreased during the time taken to complete the fifty lever presses. The injection of a low cocaine dose was not enough to raise the cocaine level to the peak levels resulting from the previous injection (Fig. 2C). As cocaine level continued to decrease while rats continued lever pressing, inter-injection interval increased and self-administration behavior on FR50 schedule eventually extinguished (Fig. 2C).

## Discussion

According to operant behavior theory, higher doses of cocaine should elicit more reinforcement and increase the rate of responding. Hence, the ascending limb of the dose–response function is the only part of the dose–response function that is consistent with cocaine’s reinforcing effects^2,20^. The present study appears to be consistent with many previous reports^2^ that at low cocaine unit doses there is an increase in the rate of lever pressing behavior and the number of self-infusions. However, this was observed only when the rats were self-administering cocaine under an FR50 schedule and not an FR1 schedule. This effect is consistent with previous reports of the effect of high FR schedules and extended time out periods^8^. Indeed, the FR50 effectively acts as a time-out period as it takes time to complete the 50 active lever presses.

As the unit doses increased, the proportion of sessions where the rats completed all the injections on FR50 increased until they could also complete all of the allowed injections. This resulted in a function analogous to the previously reported ascending limb. This apparent ascending limb on FR50 was investigated further by looking at the number of injections over a fixed time as is typical for most studies. It should be noted that in FR50 sessions where all injections were completed, the rate of injections only decreased as a function of unit dose. Importantly, in sessions where rats failed to complete all allowed injections on FR50, there was an increase in the rate of injections at the lower unit doses. This increase in the rate of injections at lower unit doses was also apparent when all of the data at a particular unit dose were combined. This demonstrates that the ascending limb of the dose-rate function is a product of combining sessions in which rats complete or fail to complete the allowed number of injections. Obviously, this would be the same if the number of responses per 1 or 3 h was presented. This is consistent with previous suggestions that the ascending limb represents an artifact that results from the averaging of initial periods with high, likely maximal, rates of responding and later periods with low, likely zero, rates of responding^21–25^. As the unit dose increases the increased duration of responding would be averaged with shorter periods of no activity.

A mechanism for the reduced probability of completing all permitted injections on the FR50 schedule at the lower unit doses is provided by the compulsion zone theory of cocaine self-administration^13^. Cocaine-induced lever pressing behavior occurred only when cocaine levels were above the priming/remission threshold and below the satiety threshold, termed the compulsion zone^13^. It can be seen that at low cocaine unit doses with restricted access to cocaine, in this case a FR50 schedule, the cocaine delivery is inadequate to maintain cocaine levels within the compulsion zone for long periods because the rate of cocaine elimination exceeds the rate of input. As the unit dose increases, the cocaine delivery increases and cocaine levels remain in the compulsion zone for longer durations. Once cocaine delivery is sufficient to maintain cocaine levels indefinitely within the compulsion zone, rats can maintain self-administration behavior indefinitely. At this point the maximum rate of cocaine delivery is possible. At higher unit doses, the rate of cocaine self-administration is limited by the time that cocaine levels are above the satiety threshold. Therefore, the ascending limb represents an artifactual increase in the probability that cocaine levels will be maintained within the compulsion zone^21–25^.

It is possible that the duration of injection across the range of unit doses with the concomitant change in time-out period may be confounded with the effect of dose. However, this study directly compared the same unit dose on the FR1 and FR50 schedules. Therefore, the duration and volume of injection were constant during a session and any inability to complete the required number of presses was due to the schedule.

Although the ascending limb of the dose response curve of cocaine self-administration is an experimental artifact that can be explained in terms of the compulsion zone theory, it should be noted that there is a more fundamental difference between the psychological theories and our pharmacokinetic/pharmacodynamic interaction approach. The psychological models assume that the magnitude of cocaine reinforcement is a graded pharmacodynamic response that will increase as a function of the dose up to a maximum when all of the relevant receptor population(s) approach saturation. In contrast, maintained self-administration or lever pressing occurs when cocaine levels fall to the satiety threshold. Consequently, the satiety threshold represents a fixed magnitude of response, and the dose–response relationship represents a dose-*duration* of response^18^, as first postulated by Levy and Nelson^26^.

In conclusion, the compulsion zone theory assumes a cocaine dose-duration of response function, it does not predict that there will be any ascending limb of the dose–response function. The present study is consistent with the compulsion zone theory. It is concluded that the ascending limb of the cocaine dose–response function is an experimental artifact resulting from averaging data from sessions where rats completed or failed to complete the allowed number of injections. This depends on maintaining cocaine levels in the compulsion zone until all injections are completed. The probability of this decreases on high FR requirements and low unit doses. Data from self-administration studies that restrict the delivery of cocaine with schedules and/or time-out periods and low cocaine unit doses should be interpreted with caution.

## Methods

### Animals

Male Sprague Dawley rats (n = 5) initial weight 200–225 g or 350–550 g over the duration of studies from Envigo (Indianapolis) were housed individually on a 14/10 h light/dark cycle with food and water available ad libitum. All studies were conducted in accordance with the National Institutes of Health *Guide for the Care and Use of Laboratory Animals* and under a protocol approved by the Institutional Animal Care and Use Committee at the University of Cincinnati, and reported in accordance with ARRIVE guidelines.

### Surgery

Rats were surgically implanted with an indwelling catheter into the right jugular vein under isoflurane anesthesia. If re-catheterization was required, catheters were placed in the left jugular and then the femoral veins as needed throughout the study. Buprenorphine (0.03 mg/rat s.c.) was administered post-surgery for pain control and gentamycin (25 mg/rat s.c.) for 3 days was used to prevent infection following surgery^27^. The catheter was flushed with heparin solution (100 units/mL in saline) once a day for the first 5 days after surgery.

### Cocaine self-administration training

Beginning at least 5 days after surgery, rats were trained to self-administer cocaine HCl (provided by the National Institute on Drug Abuse) using a fixed ratio (FR1) schedule with a time-out period equal to the injection time or 5 s, whichever was longer. Rats were weighed and flushed with 0.5 mL of heparin solution (100 units/mL in saline) immediately prior to each self-administration session. Self-administration sessions began from 9 to 10 am. Rats were placed in isolated chambers equipped with both an active and an inactive lever. The standard unit dose for training was 3 µmol/kg of cocaine HCl (40 µmol/mL in 0.9% sterile saline with 1 unit heparin/mL solution). Presses on the active lever caused activation of the pump and resulted in injection. The rate of injection of the cocaine solution was 4 µL/s. A cue light illuminated for the duration of the time out accompanied pump activation. Presses on the inactive lever had no consequences. Presses on the active lever during time-out, and all presses on the inactive lever were recorded. Detailed protocol for cocaine self-administration training and the real time computation of cocaine level can be found^28^. Rats had access to cocaine for 3 h a day, 5 days a week. The training was considered complete when rats met the criterion for acquisition of stable self-administration of the 3 µmol/kg of cocaine dose, that is less than ± 10% variance (standard deviation, SD) in the mean inter-injection interval between 3 consecutive daily sessions. After acquisition of self-administration behavior, rats were allowed to self-administer cocaine over a range of unit doses on FR1 schedule in daily sessions for 3 weeks. During the sessions rats first self-administered 2 loading injections of 3 µmol/kg cocaine. Next, they self-administered a fixed number of injections of the first dose followed by a fixed number of injections of the second dose. Lastly, rats entered the extinction or unloading phase where access to cocaine was terminated and active lever presses were recorded but had no consequence. The session was determined complete when 30 min had elapsed since the last active lever press that occurred during unloading. The doses were 0.3 µmol/kg, 0.75 µmol/kg, 1.5 µmol/kg, 3 µmol/kg, 6 µmol/kg, 12 µmol/kg, and the fixed number of injections for each dose was 75, 50, 25, 15, 10, and 5 respectively. The injection time ranged from 0.5 to 42 s, and the volume of solution injected ranged from 0.0021 to 0.17 mL depending on the unit dose and weight of the rat. The inter-injection intervals were measured and the cocaine level at the time of each lever press was calculated for each session.

### FR1/FR50 sessions at different cocaine doses

After cocaine self-administration on FR1 schedule, rats maintained responding under FR schedules of cocaine presentation greater than FR1 starting with FR 5, 10, 20 and then 50. These sessions consisted of either 2 (3 µmol/kg unit dose) or 4 (1.5 µmol/kg unit dose) self-administered cocaine loading injections, followed by 15 injections (3 µmol/kg unit dose) on FR1 and then the FR schedule greater than FR1. Then access to cocaine was terminated at the end and unloading lever presses were recorded but had no consequence. There were 1 to 3 sessions for FR1/FR5, FR1/FR10, and FR1/FR20 sessions for each rat. The differences in inter-injection intervals across this range of FR requirements were insufficient to reliably observe the ascending limb. Next, daily FR1/FR50 sessions were performed at 0.3 µmol/kg, 0.5 µmol/kg, 0.75 µmol/kg, and 1.5 µmol/kg cocaine unit doses and the unit dose to be run on a day was randomly determined. Then, FR1/FR50 sessions were performed at the 0.1 µmol/kg unit dose in order to observe evidence of an ascending limb at the FR1 schedule. There were 3 FR1/FR50 sessions at each cocaine unit dose for each rat. The FR1/FR50 phase of the study with 6 unit doses, with 3 sessions per dose, and 4 sessions per week was conducted over 5 weeks. The unit dose was always the same during the FR1 and FR50 part of a FR1/FR50 session and all FR1/FR50 sessions started with 4 self-administered loading injections of 1.5 µmol/kg unit dose.

Typical experiments calculate the frequency of responding as the total number of responses per session that typically lasts 1–3 h^2^. Because of the additional time required to complete the 50 presses, inter-injection intervals were expected to be longer at FR50 compared to FR1, and fixing the session time insures a slower rate of injections on FR50. Another approach used here is to measure the probability of completing a set number of presses with no time constraints. So, the number of injections on each FR1 and FR50 schedule was limited and was different for each unit dose. The number of injections was fixed to 225, 75, 60, 50, 25, and 15 for 0.3, 0.5, 0.75, 1.5, and 3 µmol/kg dose respectively. The number of injections at a unit dose was decided such that rats are allowed to self-administer a maximum total of 71 ± 7.4 µmol/kg cocaine in a session. When rats self-administered all allowed injections at regular intervals, the session ended with the unloading phase that is when access to cocaine was terminated and lever presses had no consequences.

At the 4 lowest unit doses, rats were observed to not maintain self-administration behavior on FR50 in some sessions. These sessions were considered to have ended when 10.7, 12, 13.3, and 15 min had elapsed since the last self-administered injection at the 0.1 µmol/kg, 0.3 µmol/kg, 0.5 µmol/kg and 0.75 µmol/kg cocaine unit dose, respectively. After these sessions ended, a programmed injection of 1.5 µmol/kg cocaine dose was given. This typically resulted in reinstatement of lever pressing behavior and self-administration. This indicated that lever pressing behavior had ceased because cocaine levels had fallen below the compulsion zone.

Rats were on an FR1 cocaine self-administration (2 loading doses of 3 µmol/kg, 75 doses of 0.3 µmol/kg and 15 doses of 3 µmol/kg cocaine followed by unloading) session every Monday throughout the course of the study. Rats were on a FR1/FR50 session Tuesday-Friday, and the order of unit dose used was random. There were 3 sessions per unit dose for each rat.

### Real time calculation of cocaine levels

The cocaine level at the time of each lever press was calculated as described previously^28^. Briefly, the cocaine level was calculated every second by a non-compartmental pharmacokinetic model that considers each cocaine injection amount and assumes first-order elimination with a half-life of 500 s.

### Statistical methods

For each measure, the values from all the sessions from a rat were used to calculate a mean for the rat. Next, the mean values of the rats were used to calculate a final mean and standard error of the mean displayed in the graphs. The number of injections at each unit dose were normally distributed. Welch’s t-test was applied to compare the number of injections on FR1 and FR50 schedule at 0.1 µmol/kg cocaine unit dose as the data did not pass the Brown-Forsythe test for equal variance. Student’s t-test was applied to compare the number of injections on FR1 and FR50 schedule at 0.3, 0.5 and 0.75 µmol/kg cocaine unit doses as these data had equal variance.

### Supplementary Information

Supplementary Information.

## Data Availability

The data sets generated during and/or analyzed during the current study are available from the corresponding author on reasonable request.

## Abbreviation

FR: Fixed ratio
